# Evidence for a lack of inotropic and chronotropic effects of glucagon and glucagon receptors in the human heart

**DOI:** 10.1186/s12933-023-01859-8

**Published:** 2023-05-30

**Authors:** Ramón Aranda-Domene, Esteban Orenes-Piñero, José María Arribas-Leal, Sergio Canovas-Lopez, Jesús Hernández-Cascales

**Affiliations:** 1 Department of Cardiovascular Surgery, Hospital CSV Arrixaca, El Palmar, 30120 Murcia, Spain; 2 Proteomic Unit, Laboratorio Investigación Biosanitaria, Av.Buenavista, 32, El Palmar, 30120 Murcia, Spain; 3 Department of Pharmacology, Faculty Medicine, Edificio LAIB, University of Murcia., 6ª Planta. Av. Buenavista, 32, El Palmar, 30120 Murcia, Spain

**Keywords:** Glucagon receptor, Human heart, Chronotropism, Inotropism

## Abstract

**Background:**

Glucagon is thought to increase heart rate and contractility by stimulating glucagon receptors and increasing 3′,5′-cyclic adenosine monophosphate (cAMP) production in the myocardium. This has been confirmed in animal studies but not in the human heart. The cardiostimulatory effects of glucagon have been correlated with the degree of cardiac dysfunction, as well as with the enzymatic activity of phosphodiesterase (PDE), which hydrolyses cAMP. In this study, the presence of glucagon receptors in the human heart and the inotropic and chronotropic effects of glucagon in samples of failing and nonfailing (NF) human hearts were investigated.

**Methods:**

Concentration‒response curves for glucagon in the absence and presence of the PDE inhibitor IBMX were performed on samples obtained from the right (RA) and left atria (LA), the right (RV) and left ventricles (LV), and the sinoatrial nodes (SNs) of failing and NF human hearts. The expression of glucagon receptors was also investigated. Furthermore, the inotropic and chronotropic effects of glucagon were examined in rat hearts.

**Results:**

In tissues obtained from failing and NF human hearts, glucagon did not exert inotropic or chronotropic effects in the absence or presence of IBMX. IBMX (30 µM) induced a marked increase in contractility in NF hearts (RA: 83 ± 28% (n = 5), LA: 80 ± 20% (n = 5), RV: 75 ± 12% (n = 5), and LV: 40 ± 8% (n = 5), weaker inotropic responses in the ventricular myocardium of failing hearts (RV: 25 ± 10% (n = 5) and LV: 10 ± 5% (n = 5) and no inotropic responses in the atrial myocardium of failing hearts. IBMX (30 µM) increased the SN rate in failing and NF human hearts (27.4 ± 3.0 beats min^**−1**^, n = 10). In rat hearts, glucagon induced contractile and chronotropic responses, but only contractility was enhanced by 30 µM IBMX (maximal inotropic effect of glucagon 40 ± 8% vs. 75 ± 10%, in the absence or presence of IBMX, n = 5, P < 0.05; maximal chronotropic response 77.7 ± 6.4 beats min^**−1**^ vs. 73 ± 11 beats min^**−1**^, in the absence or presence of IBMX, n = 5, P > 0.05). Glucagon receptors were not detected in the human heart samples.

**Conclusions:**

Our results conflict with the view that glucagon induces inotropic and chronotropic effects and that glucagon receptors are expressed in the human heart.

**Supplementary Information:**

The online version contains supplementary material available at 10.1186/s12933-023-01859-8.

## Introduction

Glucagon is a polypeptide hormone that is produced and secreted by alpha cells of the pancreatic islets of Langerhans, and it increases glucose production and counteracts the effects of insulin to maintain normoglycaemia in the fasting state. In addition to its metabolic effects, glucagon is considered to be a cardiostimulatory agent that increases heart rate and contractility [1, 2]. The mechanism responsible for these effects involves the stimulation of cardiac glucagon receptors via Gs protein stimulation, which causes adenylyl cyclase activation and an increase in 3′,5′-cyclic adenosine monophosphate (cAMP) production in the myocardium [2]. The first report on the inotropic and chronotropic effects of glucagon was presented by Farah and Tuttle in 1964 and showed an increase in heart rate and contractility after administering glucagon to failing and nonfailing dog hearts [3]. Further experimental work supported the chronotropic and inotropic effects of glucagon and showed interspecies differences in these effects [3, 4]. Moreover, glucagon seems to induce differing effects by heart region, and there is a more marked response at the ventricular level than at the atrial level [5–7].

Soon after the chronotropic and inotropic effects of glucagon were observed in animal studies, and under the assumption that those effects also occur in humans, glucagon was administered to patients suffering from cardioinhibitory conditions such as heart failure or cardioinhibitory drug overdose, mainly β-adrenoceptor blocking drugs [8]. However, conflicting results have been obtained, and the clinical efficacy of glucagon in these cases has not yet been confirmed [9, 10]. Additionally, studies of human heart samples have produced contradictory results. In vitro studies have shown a negative inotropic effect, no inotropic effect, or only a weak inotropic effect of glucagon on samples of human myocardium [11, 12]. The reason for these contradictory effects is unknown. It has been suggested that these differences may be related to the degree of heart dysfunction [11, 13] but they may also be due to differences in tissue cyclic nucleotide phosphodiesterase (PDE) enzyme activity. Indeed, PDE breaks down cAMP into the chemically inactive product 5’AMP [14] and can blunt or nullify cAMP-dependent inotropic effects, including those of glucagon [6, 15], but this hypothesis has not been tested in the human myocardium. Although the cardiostimulatory effect of glucagon on the human heart has not been established, American and European clinical guidelines recommend glucagon as an antidote to counteract cardiac depression in the case of β-adrenoceptor blocking overdose [16–18]. The purpose of the present study was to investigate whether glucagon exerts direct inotropic or chronotropic effects on samples obtained from the human myocardium and human sinoatrial node (SN) tissue in vitro. We also examined glucagon in rat hearts, where it is known to produce these effects [6, 19]. To examine possible regional differences in the effects of glucagon on the human heart, as has been reported in rats and dogs [5–7], the inotropic effect of glucagon was examined in tissues obtained from the right and left atria, as well as from the right and left ventricles. Since the inotropic effect of glucagon has been suggested to depend on the degree of cardiac dysfunction [11, 13], the effect of glucagon was studied in samples obtained from failing and nonfailing myocardia. In parallel to the inotropic and chronotropic effects of glucagon, the presence of glucagon receptors in the human heart was also investigated.

## Methods

### Tissue collection

Human cardiac tissues were obtained from the nonfailing hearts of multiorgan donors that were not used for transplantation and from the explanted hearts of patients with end-stage myocardial failure who underwent heart transplantation at the Hospital CSV Arrixaca of Murcia (Spain). Nonfailing hearts used in this study were in asystole for 83 ± 23 min (n = 5) before explantation, while organs other than the heart were retrieved for transplantation. Failing hearts were not in asystole before explantation. The characteristics of the heart donors are described in Table 1. Immediately after explantation, parts of the free wall of the atrial (right, RA and left, LA) and ventricular (right, RV and left, LV) myocardium, as well as from the portion of the right atrium attached to the entrance of the superior vena cava, were dissected. After being removed, these tissues were placed in oxygenated cool Tyrode solution containing 136.9 mmol/L NaCl, 5.0 mmol/L KCl, 1.8 mmol/L CaCl_2_, 1.5 mmol/L MgCl_2_, 0.4 mmol/L H_2_PO_4_, 11.9 mmol/L NaHCO_3_, and 5.0 mmol/L dextrose and transported to the laboratory (approximately 15 min). To investigate the inotropic effects of glucagon, RA, LA, RV and LV tissues were dissected to yield trabecular strips measuring 4–6 mm in length and 0.4–0.8 mm in diameter. The chronotropic effects of glucagon were investigated in SN tissue obtained from the right atrial wall where the crista terminalis meets the superior vena cava. To better find the SN region, we followed the SN artery trajectory by carefully removing the epicardium and subepicardial connective tissue from the external surface of the right atrium. The atrial wall, to which the SN artery connects, contains the SN [20]. This portion of the atrial wall, as well as the attached pectinate muscle, was dissected. Contraction of the pectinate muscle when stimulated by SN tissue was recorded as the measure of the SN rate. In addition to functional identification, we aimed to identify SN tissue by determining the level of the hyperpolarization-activated cyclic nucleotide-gated 1 (HCN1) channel, which is predominantly located in SN tissue [21].

**Table 1:** Characteristics of the heart donors

Heart No	Age	Sex	HF	TABTR (mins.)	Diagnosis	Treatment
1	55	F	+	−	HCM, HT,AF	Acenocumarol, Bisoprolol, Sacubitril/Valsartan, Spironolactone, Furosemide
2	64	F	+	−	HCM, AF, Hypothyroidism	Acenocumarol, Bisoprolol, Bumetanide, Spironolactone, Levothyroxine
3	41	M	+	−	Autoinmune myocarditis	Dobutamine, Levosimendan
4	55	M	+	−	IDC, OSA, Hypothyroidsm	Bisoprolol, Eplerenone, Furosemide, Diazepam, Sacubitril/Valsartan, Levothyroxine
5	67	M	+	−	FDM, Stroke, CKD, AF	Furosemide, Bisoprolol, Eplerenone, Sacibitril/Valsartan, Dabigatran
6	70	F	−	20	TBI	−
7	63	M	−	90	TBI, Prolapsed disc, DLP	Tramadol. Paracetamol, Statins
8	69	F	−	120	CKD, HT, DLP	Zolpidem, Furosemide, Doxazosin, Amlodipine, Bisoprolol, Statins
9	71	M	−	25	Subaracnoid hemorrage, AF	Acenocumarol, Bisoprolol
10	42	M	−	160	TBI, Smoker	−

*HF* Heart Failure, *TABTR* Time before tissues retrieval, *F* Female, *M* Male, *HT* Hypertension, *AF* Atrial Fibrillation, *DLP* Dislipemia, *HCM* Hypertrophic Cardiomyopathy, *CKD* Cronic Kidney disease, *OSA* Obstructive Sleep apnoea, *FDM* Familiar dilated cardiomyopathy, *TBI* Traumatic brain injury; *IDC* Ischaemic dilated cardiomyopathy

We also evaluated the inotropic and chronotropic effects of glucagon on rat hearts. For this purpose, Sprague–Dawley rats (250–300 g, both sexes) were stunned and exsanguinated. The chest was opened, and the heart was rapidly removed and placed in Tyrode solution saturated with 95% O2 and 5% CO2. The right atrium and the free wall of the right ventricle were excised, and strips of the right ventricle (1.5 mm wide, 10 mm long, and 1 mm thick) were obtained. All procedures were performed in the presence of Tyrode solution.

### Sample setup and construction of concentration–response curves

To investigate inotropic effects, trabeculae from human myocardium (RA, LA, RV and LV) or strips of the rat right ventricle were mounted vertically between two platinum electrodes in a 30 ml double-walled organ bath containing Tyrode solution at 37 °C, pH 7.4, and 95% O_2_ plus 5% CO_2_. The samples were electrically stimulated (Grass SD-9 stimulator) at a frequency of 1 Hz for 3 ms with supramaximal (threshold + 25%) voltage. A length–force curve was obtained, and the tissues were left at the length associated with the maximum developed force [6, 15].

The chronotropic effect of glucagon was studied in human SN tissue that was obtained as previously described, as well as in isolated rat right atria. The tissues were mounted vertically in a 30 ml double-walled glass chamber filled with Tyrode solution, gassed continuously with 95% O_2_ and 5% CO_2,_ and maintained at pH 7.4 and 37 °C. A preload tension of 0.5 gm. was applied. Under these conditions, human SN samples (Additional file 1), as well as rat right atria, started beating spontaneously. Contractions were measured using a Grass FT-03 force‒displacement transducer (Quincy, MA, USA) and displayed on a computer screen using a Stemtech amplifier (Stemtech Inc., Houston, Texas) and ACODAS software (DATAQ Instruments, Inc., Akron, Ohio). The tissues were allowed to equilibrate for 45–60 min in Tyrode solution.

After equilibration, cumulative concentration‒response curves to glucagon (Novo Nordisk Pharma S.A. Madrid, Spain), were determined in human and rat tissues by increasing the concentration stepwise by 0.5 log units.

To ascertain the role of PDE in regulating glucagon responses, a second cumulative concentration‒response curve was obtained for glucagon in the presence of the nonselective PDE inhibitor 3- isobutyl-1-methylxanthine (IBMX) [14] (Sigma/Aldrich, Madrid, Spain). The samples were washed and left to stabilize during an additional period of 30 min, and then 30 μM IBMX was applied. This concentration of IBMX effectively inhibited the predominant PDE activity in human and rat hearts [22, 23]. IBMX was left in contact with the tissue for 15 min before the construction of a second concentration–response curve for glucagon. Drugs were added to the organ bath in volumes less than or equal to 0.1 ml. The experiments of contractility (human and rat tissues) and chronotropism (rat right atrial and human SN tissue) were finalized with the addition of 9 mM CaCl_2_ and 10 µM noradrenaline (Sigma/Aldrich, Madrid, Spain), respectively, to determine the response capacity of the samples. Changes in contractile force are expressed in mN and as percentages with respect to the basal control contraction amplitude. The frequency is expressed as beats min^**−1**^, and the results are expressed as differences with respect to the basal rate.

### Real-time PCR

Samples from the right and left atria, right and left ventricles and SNs were carefully dissected. Quantitative PCR was performed using QuantStudio 5 (Applied Biosystems, Thermo Fisher Scientific, Massachusetts, USA). Total mRNA was extracted from heart tissue using TriPure Isolation Reagent (Roche, Paris, France), and 1 μg of RNA was used for the reverse transcription reaction (iScript cDNA Synthesis kit. Bio-Rad, CA, USA). The reactions were carried out in a final volume of 5 μl containing 300 nM primers and 1 μl of cDNA using a SYBR Premix Ex Taq (Tli RNaseH Plus) kit (Takara BioInc., Göteborg, Sweden). The samples were subjected to the following conditions: 30 s at 95 °C, 40 cycles (10 s at 95 °C, 30 s at 60 °C), and a melting curve at 60–95 °C with a slope of 0.1 °C/s. The reference gene glyceraldehyde-3-phosphate dehydrogenase (GAPDH) was used as the endogenous control for quantification (KiCqStart Primers, Merck, Darmstadt, Germany). The resulting values are expressed as the relative levels with respect to the control levels (2^−ΔΔCT^). Human liver tissue (“Biobanco Región de Murcia”, national register number B.0000859) was used as a positive control for glucagon receptor gene expression.

Specific primers for gene level analysis, as well as accession numbers and amplicon lengths, are shown (Additional file 2: Table S2).

### Tissue samples for western blotting

Total protein was extracted from heart samples (right and left atria, right and left ventricle, and SN). Briefly, 100 mg of frozen tissues was disrupted in a polytron homogenizer using radioimmunoprecipitation assay lysis buffer with protease and phosphatase inhibitor cocktails and quantified with a Bradford assay using bovine serum albumin as a standard (Protein Assay Kit, Bio-Rad, Hercules, CA). Total protein extracts (30 µg) were mixed with 5 × sodium dodecyl sulfate (SDS) sample buffer (62.5 mM Tris–HCl, pH 6.8, 2% SDS, 10% glycerol, 5% β-mercaptoethanol, and 0.005% bromophenol blue) and resolved by SDS–polyacrylamide gel electrophoresis on 10% acrylamide gels. Proteins were detected immunologically following electrotransfer to polyvinylidene fluoride membranes (Millipore, Bedford, MA) that were activated with methanol. The membranes were blocked with 5% nonfat dry milk in phosphate-buffered saline (PBS) and 0.1% Tween-20 for 1 h at room temperature and incubated overnight at 4 °C with the following primary antibodies: anti-glucagon receptor (rabbit polyclonal, 1:500 dilution, MyBioSource Inc. San Diego, CA) and anti-HCN1 (rabbit polyclonal, 1:1000 dilution, Sigma/Aldrich, Madrid, Spain). The blots were washed three times for 10 min each in PBS and 0.1% Tween-20 and incubated with horseradish peroxidase-conjugated goat anti-rabbit IgG secondary antibodies (1/5000 dilution) (Arigo Biolaboratories, Hsinchu City, Taiwan) for 1 h at room temperature. The blots were developed using a peroxidase reaction with an enhanced chemiluminescent immunoblotting detection system (ECL-Plus, GE Healthcare, Little-Chalfont, Buckinghamshire, UK). Antibodies were accepted when they exhibited a single predominant band at the expected molecular weights. α-Tubulin (mouse monoclonal, 1:10,000 dilution, Proteintech, Deansgate, Manchester, UK) was used as the loading control. Band intensity was determined by densitometry using the program Image Quant TL Plus (General Electric, USA).

### Statistical analysis

The results are expressed as the mean values ± standard errors. Concentration‒response curves were fitted with a nonlinear regression sigmoidal concentration‒response curve, variable slope, and –log EC_50_, and the maximal effect (E_**max**_) values were estimated from the concentration‒response curves depicted. Concentration‒response curves and statistical analyses were performed with GraphPad 5 Software, Inc. (San Diego, CA, USA). Statistical significance was determined by paired or unpaired Student`s t test, Wilcoxon test and parametric binomial analysis as appropriate. A P value ≤ 0.05 was considered statistically significant.

## Results

### Inotropic effects

**Fig. 1:**
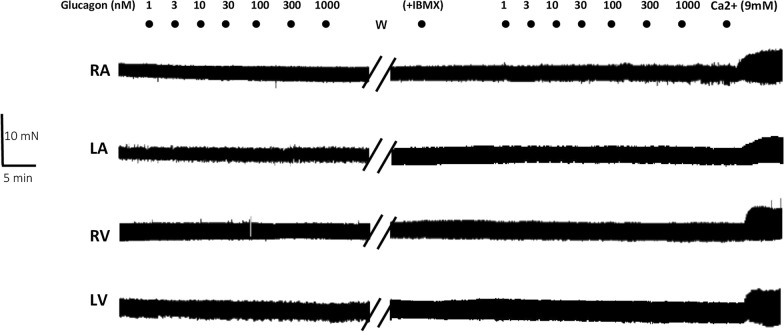
Representative traces showing that glucagon did not exert inotropic effects on the failing human myocardium. Increasing concentrations of glucagon (1–1000 nM) were added to trabecular strips obtained from the right atrium (RA), left atrium (LA), right ventricle (RV) and left ventricle (LV) obtained from a failing heart. After being washed (W), the samples were allowed to stabilize for 30 min, and then the nonselective phosphodiesterase inhibitor IBMX (30 μM) was applied and left in contact with the tissue for 15 min. Afterwards, increasing concentrations of glucagon (1–1000 nM) were again applied to all samples, and again, no inotropic effect was observed. Experiments were finalized with the addition of 9 mM CaCl_2_ to determine the responsiveness of the samples

**Fig. 2:**
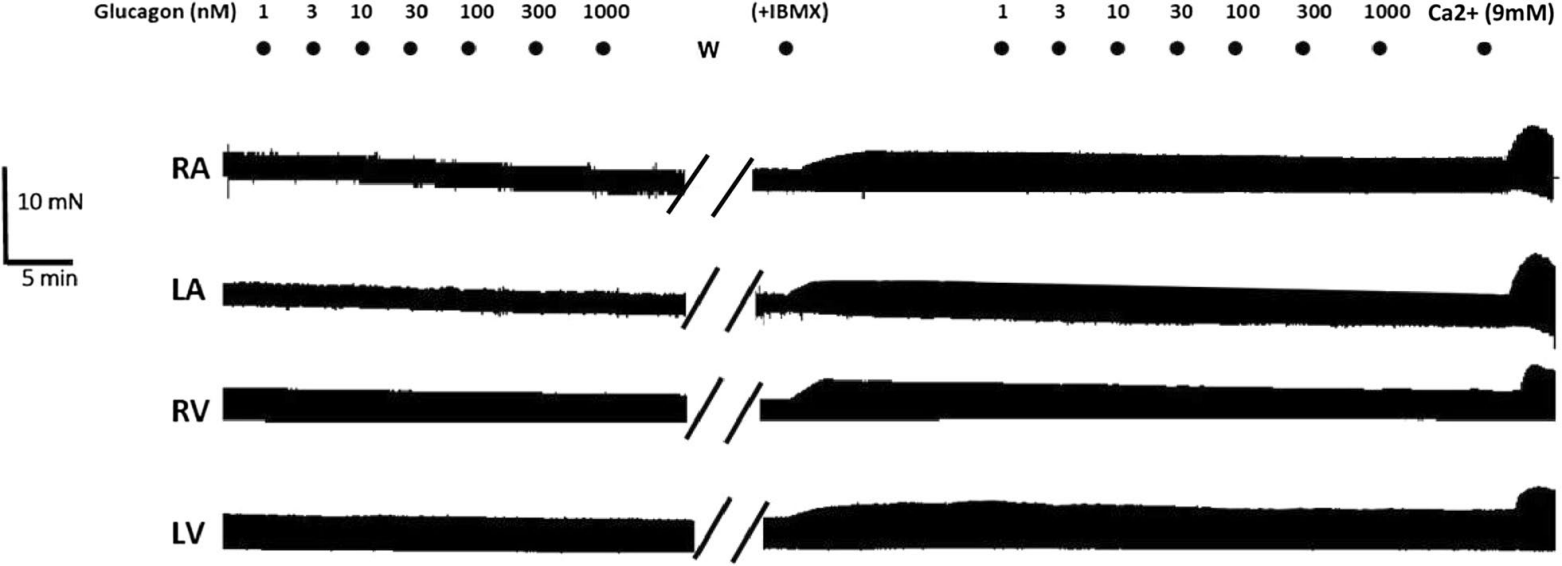
Representative traces showing that glucagon did not exert inotropic effects on the nonfailing human myocardium in the absence or presence of IBMX. Further details are provided in the legend to Fig. 1

**Fig. 3:**
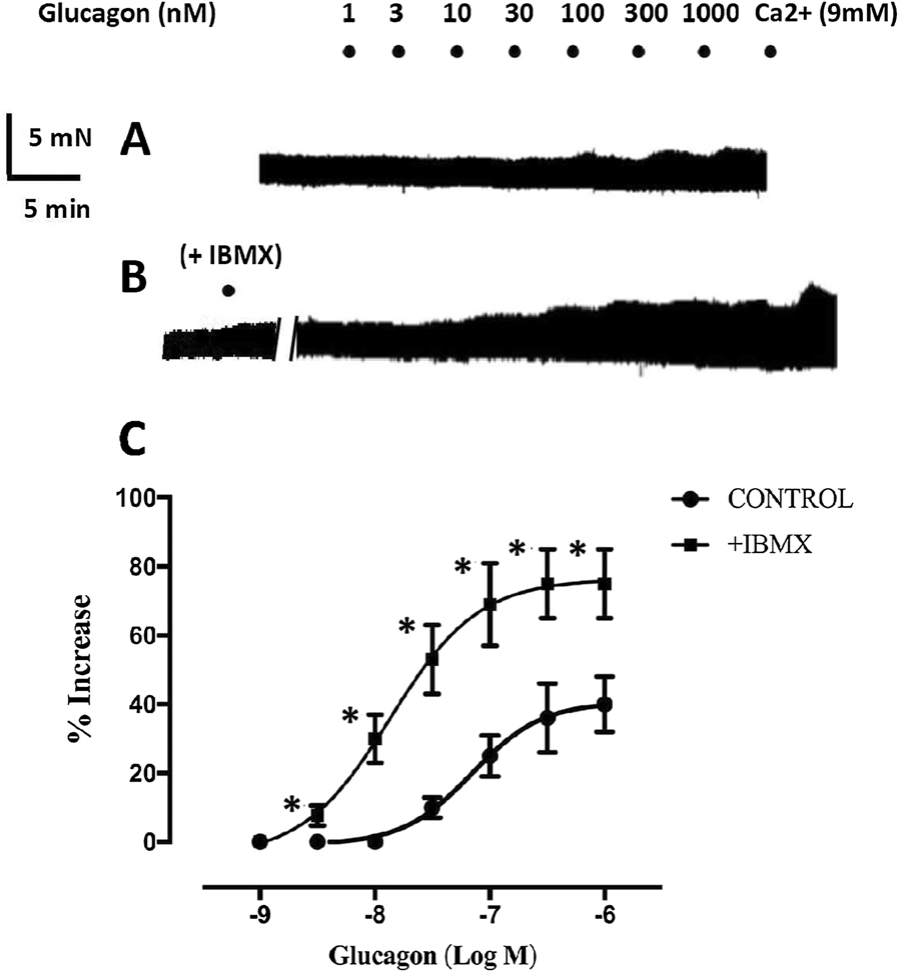
Representative traces of a strip of the right ventricle of a rat heart showing **A** the contractile effect of glucagon (1–1000 nM) alone and, after washing, in the presence of 30 µM IBMX **B**, which increased contractility on its own and enhanced the inotropic effect of glucagon. **C** Cumulative concentration‒response curves showing the inotropic effect of glucagon alone (●) and in the presence of 30 µM IBMX (■). Contractility in the presence of IBMX was used as the baseline value for the second concentration‒response curve of glucagon. Inotropic responses are expressed as percent contractility increase compared to baseline. Each point represents the mean value ± SEM (vertical bars) of 5 experiments. *P < 0.05 compared to baseline (paired Student’s t test)

The effect of glucagon on cardiac contractility in atrial (RA and LA) and ventricular (RV and LV) human myocardial tissue is shown in Figs. 1 and 2. Glucagon did not exert inotropic effects in trabeculae obtained from explanted failing (Fig. 1) and nonfailing human hearts (Fig. 2). To determine whether the inhibition of PDE activity unmasked inotropic responses to glucagon, a second concentration‒response curve for glucagon was established in the presence of the PDE inhibitor IBMX (30 µM). This concentration of IBMX alone increased contractility mainly in myocardial tissue obtained from nonfailing hearts. No response to IBMX was observed in atrial trabeculae from failing hearts, but IBMX induced a robust inotropic response in atrial tissues obtained from nonfailing hearts (Additional file 3: Figure S3). Ventricular myocardium from failing and nonfailing hearts responded to IBMX by increasing contractility, but the inotropic response of ventricular trabeculae from nonfailing hearts was higher than that from failing hearts (Additional file 3: Figure S3). In the presence of IBMX, glucagon did not exert inotropic effects on failing or nonfailing human myocardium (Figs. 1 and 2). The addition of calcium increased contractility in atrial and ventricular tissues obtained from failing or nonfailing hearts (Additional file 4: Figure S4). In contrast to the human myocardium, in the rat ventricle, glucagon induced a positive concentration-dependent inotropic response (Fig. 3A). IBMX (30 µM) increased rat ventricular contractility by 25 ± 7% (n = 5), shifted the concentration‒response curve of glucagon to the left (Fig. 3B–C), changed its –log EC50 (7.1 ± 0.08 for glucagon alone and 8.1 ± 0.1 (n = 5) in the presence of IBMX; P < 0.05, paired Student’s t test) and increased the E_**max**_ value of glucagon (40 ± 8% in the absence and 75 ± 10% (n = 5), in the presence of IBMX; P < 0.05, paired Student’s t test).

### Chronotropic effects

The samples of human SN tissue developed spontaneous contractions 10–20 min after being mounted. The basal rate was lower in tissues obtained from nonfailing hearts than in those obtained from failing hearts (34 ± 4.7, n = 5, vs. 53 ± 4.0 beats min^**−1**^, n = 5; P < 0.05, Wilcoxon test). Glucagon did not exert chronotropic effects on samples obtained from failing or nonfailing hearts. After washing, the effects of glucagon were examined in the presence of 30 µM IBMX. This concentration of IBMX produced a similar increase in the rate of SN tissue from nonfailing and failing hearts (Additional file 5: Figure S5), which was 27.4 ± 3.0 beats min^**−1**^ (n = 10). Glucagon also failed to produce any chronotropic effects in the presence of IBMX. Experiments were terminated by adding 10 µM noradrenaline, which further increased the sinoatrial rate (69.7 ± 8.8 beats min^**−1**^, n = 10). The chronotropic effect of noradrenaline was similar in tissues obtained from nonfailing and failing hearts (Additional files: Figure S5).

**Fig. 4:**
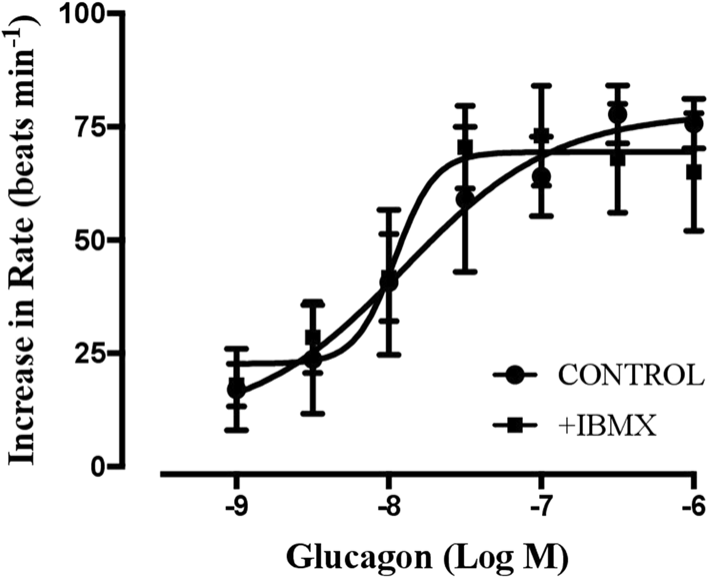
Concentration‒response curves for the positive chronotropic effects of glucagon in the absence (●) and presence of the nonselective phosphodiesterase inhibitor IBMX (30 μM, ■) in spontaneously beating rat right atria. The results are expressed as the increase in the basal rate (beats min^**−1**^). IBMX increased the beating rate by 33 ± 12 beats min^**−1**^ (n = 5). The beating rate in the presence of IBMX was used as the basal rate for the second concentration‒response curve of glucagon. Each point represents the mean value ± SEM (vertical bars) of 5 experiments. No significant differences were found when comparing any concentrations of glucagon in the absence or presence of IBMX (paired Student’s t test)

In the spontaneously beating rat right atria, glucagon induced a positive chronotropic effect, which was reversed by washing. Inhibiting PDE activity with IBMX (30 µM) increased the atrial rate by 33 ± 12 beats min^−1^ (n = 5) but did not alter the chronotropic effect of glucagon (Fig. 4). The Emax value for glucagon was 77.7 ± 6.4 beats min^−1^ in the absence of IBMX and 73.0 ± 11.1 beats min^−1^ in the presence of IBMX (n = 5, P > 0.05, paired Student’s t test). Additionally, the –log EC50 values for glucagon were not different (7.8 ± 0.4 in the absence and 7.9 ± 0.1 in the presence of IBMX; n = 5, P > 0.05, paired Student’s t test).

### The expression of glucagon receptors in human atrial and ventricular myocardium and sinoatrial node (SN) tissue

**Fig. 5:**
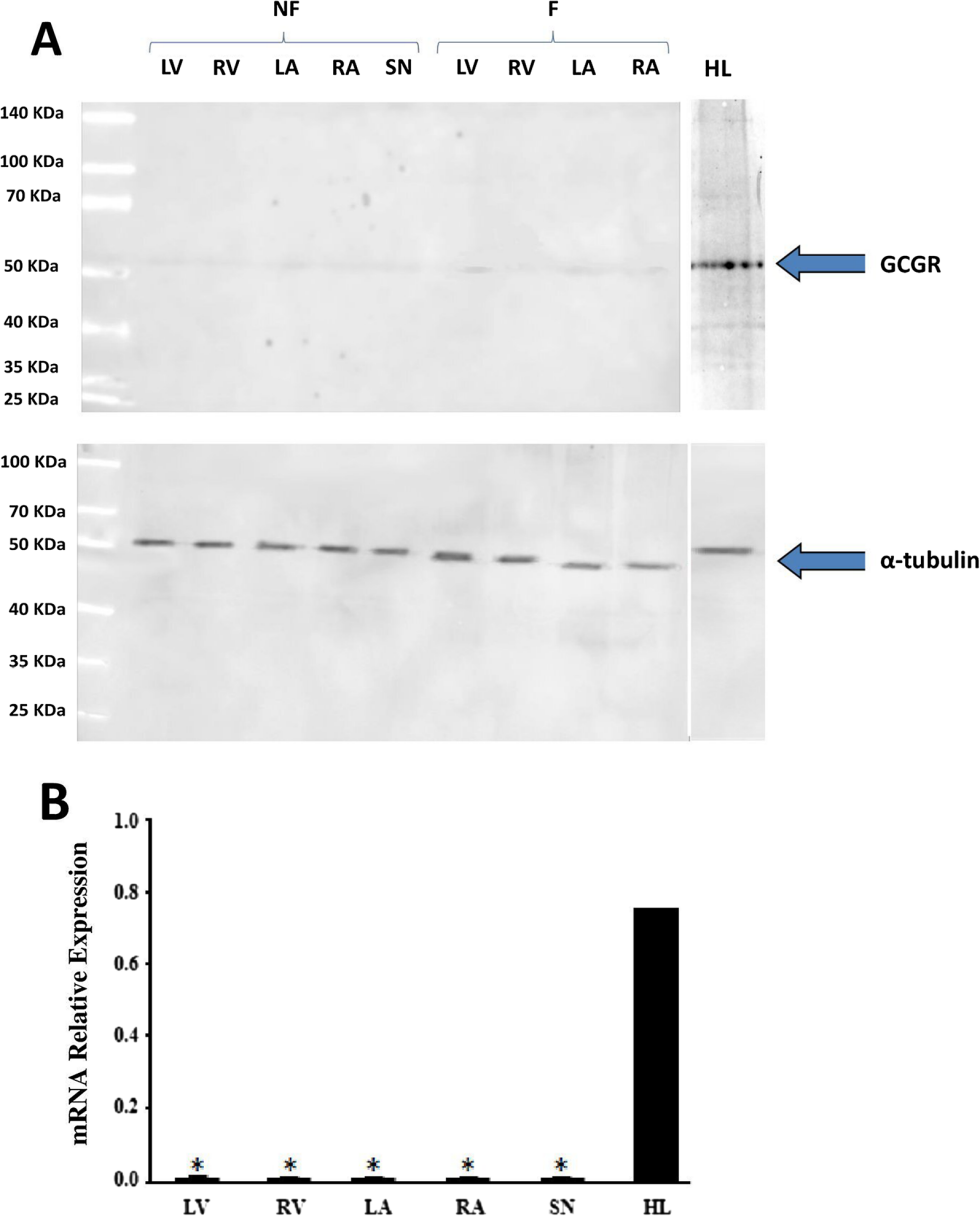
**A** Western blot analysis showing the absence of glucagon receptor expression in samples of sinoatrial node (SN), right atrium (RA), left atrium (LA), right ventricle (RV) and left ventricle (LV) from a failing (F) and a nonfailing (NF) human heart. Samples correspond to heart donors number 5 F and number 6 (NF) in Table 1. α-Tubulin was used as the loading control. Human liver (HL) was used as the positive control. Band intensity was determined with densitometry using the program Image Quant TL Plus (General Electrics, USA). **B** The absence of glucagon receptor mRNA in failing (n = 5) and nonfailing (n = 5) human myocardium samples. Samples were obtained from the sinoatrial node (SN), right atrium (RA), left atrium (LA), right ventricle (RV), and left ventricle (LV). Human liver (HL) tissue was used as the positive control. Each bar represents the combined result of samples obtained from failing and nonfailing hearts (n = 10). Only one sample of the human liver was used as the control. *P < 0.015 compared to HL (parametric binomial analysis)

Next, we examined glucagon receptor expression in the atrial and ventricular human myocardium, as well as in the human liver as a positive control. Figure 5A shows the absence of glucagon receptors in the human heart. Glucagon receptors are not expressed in any of the four cardiac chambers in failing or nonfailing human hearts. Similarly, glucagon receptors were not detected in the rest of the samples obtained from failing or nonfailing hearts (Additional file 6 Figure S6). In contrast, glucagon receptors were expressed in human liver, as expected (Fig. 5A).

We also investigated the presence of glucagon receptors in the SN. First, we aimed to identify human SN tissue by the abundance of HCN1, which is the predominant cyclic nucleotide-gated channel [21]. We also determined HCN1 levels in samples of human right ventricles for comparison and found that HCN1 expression was approximately 8 times higher in the SN than in the right ventricle (Additional file: 7 Figure S7), suggesting that the samples contained SN tissue. Glucagon receptor expression was not detected in SN tissue from failing or nonfailing hearts (Fig. 5A; Additional file 1: Figure S6).

In addition to glucagon receptor expression, we also analysed the mRNA levels of the glucagon receptor in tissues obtained from RA, LA, RV, LV, and SNs by real-time PCR, and human liver was used as a positive control. The mRNA expression of the glucagon receptor was abundant in the human liver but was not found in any cardiac tissue (Fig. 5B).

## Discussion

In contrast to the inotropic effects of glucagon in animal studies [3, 6, 7], our results indicated that glucagon did not exert a direct inotropic effect on the human myocardium. Furthermore, this study was the first to examine the direct effect of glucagon on the human SN rate, which showed no chronotropic effect on this tissue.

Glucagon is a cardiostimulatory agent that increases heart rate and contractility [1, 2]. These effects are attributed to the stimulation of glucagon receptors associated with Gs protein, which causes adenylyl cyclase activation and increased cAMP production [2], which in turn increases cardiac automaticity and contractility [24, 25]. The inotropic and chronotropic effects of glucagon have been demonstrated in isolated samples obtained from the hearts of different animal species [3, 5, 6], but whether glucagon induces these effects on the human heart has not yet been established. Soon after the cardiostimulatory effects of glucagon were observed in animals, glucagon was administered to patients with low cardiac output states under the assumption that similar effects would occur with the human heart, thereby exerting a beneficial therapeutic effect. However, conflicting results were obtained because although some clinical improvements have been observed, no effect or death has been reported after administering glucagon to these patients [8, 9, 26]. Furthermore, whether the reported beneficial effects of glucagon are due to direct inotropic and chronotropic effects on the myocardium or another mechanism is also unknown and warrants investigation. The aim of the present study was to shed some light on the direct effects of glucagon on the human heart. Moreover, the effects of glucagon on the isolated right atrium and ventricular myocardium of rat hearts were examined. In rats, glucagon was reported to produce chronotropic and inotropic effects [6, 19], and that was verified in the present study. The positive inotropic effect of glucagon on rat ventricular heart tissue is due to the activation of glucagon receptors in this tissue and subsequent Gs activation and cAMP production [2, 6]. Reducing cAMP hydrolysis by PDE inhibition enhanced glucagon-producing cAMP levels and increased the inotropic effect of glucagon on the rat myocardium [6, 27]. This is consistent with the enhancement of the inotropic effect of glucagon induced by the nonselective PDE inhibitor IBMX in rat ventricular myocardium in this study. Glucagon also induces a chronotropic effect on the isolated right atria of rat hearts, but in contrast to the inotropic effect, the chronotropic effect was not enhanced by the nonselective PDE inhibitor IBMX. This finding was consistent with previous results showing that the chronotropic effect of glucagon, as well as that of the β-adrenoceptor agonists noradrenaline or isoproterenol, were not affected by PDE inhibition [19, 28]. The reason why the chronotropic effects of cAMP-dependent agents such as β-adrenoceptor agonists or glucagon are not affected by PDE inhibition is not clear, although it has been proposed that the increased cAMP induced by these agents is compartmentalized in the pacemaker cell in a microdomain different from that where PDE is located or in a microdomain where access is difficult for PDE inhibitors [28].

In contrast to animal studies, including the rat heart results in the present study, in which the inotropic effect of glucagon has been demonstrated (3, 5, 6), evidence for a contractile effect of glucagon on the human heart is scarce and conflicting. For instance, in a pioneering in vitro study of ventricular papillary muscles obtained from 13 patients with heart failure (12 NYHA grade III-IV and 1 grade II), glucagon induced a weak positive inotropic effect on three samples, a negative inotropic effect on another three samples, and no inotropic effect on the other papillary muscles. The proposed explanation for those results was different degrees of myocardial functional impairment. Thus, glucagon induces an inotropic response in papillary muscles from patients with conserved cardiac function but not in those from patients with chronic heart failure [13]. In another study by Prasad (1972) [11] of papillary muscles obtained from patients undergoing corrective open-heart surgery, glucagon was effective in increasing the force of contraction in only 2 out of 20 papillary muscle samples. However, in another study by the same author, which was also on papillary muscles obtained from patients undergoing corrective heart-valvular surgery, glucagon not only failed to induce any inotropic effect but also tended to decrease contractility, although the decrease observed was not significant [12]. In our study, glucagon did not exert inotropic effects on any of the human tissues studied, whether they were obtained from explanted hearts of patients with advanced heart failure or from donors without heart failure. In these tissues, however, calcium induced inotropic responses. Consequently, our results do not suggest an inotropic effect of glucagon on the human myocardium, regardless of whether heart function is more or less conserved. This finding is further supported by the absence of protein or mRNA expression of glucagon receptor in atrial and ventricular human myocardium. This was consistent with recent findings that glucagon receptor mRNA expression was absent from the left ventricle (the most important cardiac chamber for maintaining adequate haemodynamic parameters) and that only traces were detected in the left or right atria and right ventricle in 2 out of 15 human hearts studied [29]. Therefore, the absence of an inotropic effect of glucagon on the human myocardium observed in this and other studies [12, 30] is most likely due to a lack of glucagon receptors rather than to impaired myocardial function, as had been suggested [13]. This view is supported by the effect of glucagon in the presence of the nonselective PDE inhibitor IBMX. Since the inotropic effect of glucagon is cAMP dependent, inhibiting cAMP hydrolysis with IBMX should have increased this effect, which was observed in rat ventricular myocardium, which is known to express glucagon receptors [6]. However, in the human myocardium, glucagon failed to induce any inotropic effect with or without the presence of IBMX. Interestingly, IBMX alone induced a robust contractile response in tissues from nonfailing hearts, but there were almost no inotropic effects on the failing myocardium. This finding was consistent with previous studies showing a reduced inotropic response to PDE inhibitors in the failing human heart, which has been attributed to an increase in signal transducing inhibitory G-proteins that may maintain reduced adenylate cyclase activity rather than changes in PDE activity [31].

Animal studies have shown regional differences in the inotropic effect of glucagon, and glucagon receptor expression was higher at the ventricular level than at the atrial level [5, 6]. However, our results indicate that this is not the case in the human heart, since differences in contractile responses to glucagon or glucagon receptor expression do not exist between atrial and ventricular myocardium.

The chronotropic effect of glucagon has been evaluated in the human SN. Samples of SN tissues from nonfailing hearts had slower rates than those obtained from failing hearts. This is probably due to prolonged asystole time in hypoxia in the nonfailing hearts while other organs were retrieved for transplantation, which would affect sinus node function and reduce its rate. In addition to inotropic effects, glucagon did not exert chronotropic effects on the human SN alone or in the presence of IBMX. In contrast, IBMX and noradrenaline produced a consistent increase in the SN rate. The lack of a chronotropic response to glucagon is consistent with the absence of glucagon receptor mRNA and protein expression in the human SN.

Taken together, the results of this study indicate that glucagon did not exert direct inotropic and chronotropic effects on the human heart. However, possible reflex mechanisms that could induce these effects cannot be excluded. Some studies [24, 32] but not others [30, 33] reported glucagon-induced increases in cardiac output, cardiac index, and heart rate in humans. Moreover, some positive results have been reported after glucagon administration to patients with low cardiac output [32, 34], symptomatic bradycardia [35], or an overdose of cardioinhibitory drugs such as beta-blockers, calcium channel blockers, or even antidepressants [35–37]. However, nearly all of these studies were conducted prior to the availability of recombinant glucagon and used standard glucagon preparations instead. The standard preparation of glucagon, which is made from mammalian pancreatic extract, contained insulin (also from pancreatic cells) until recombinant glucagon was made available in 1998 [38]. Indeed, allergic reactions to numerous glucagon samples have been shown to be directly proportional to the amount of insulin contamination in those preparations [39]. Since insulin increases human cardiac contractility [40] as well as heart rate [41] and shows efficacy in treating cardioinhibitory conditions [10], it is possible that the results obtained using these glucagon preparations may have been due to the insulin that was also present. Other reported effects of glucagon, such as vasodilation [42], catecholamine (mainly adrenaline) release from the adrenal gland [43], activation of the hypophysis–hypothalamus–adrenal axis [44], or the stimulation of sympathetic activity at the hypothalamic level [45], could have been involved in the reported cardiostimulatory effects of glucagon in humans. However, further studies are required to establish the underlying mechanisms.

## Limitations

One limitation of this study is the relatively low number of cases included. Thus, new works testing other antiglucagon receptor antibodies or comparing glucagon with other Gs protein stimulators, may help to complement the results and conclusions of this study.

## Conclusions

In conclusion, the results of this study indicate that glucagon did not exert inotropic and chronotropic effects, and there was an absence of glucagon receptors in the human heart. Even so, glucagon administration is recommended in the case of beta-blocker overdose [16, 17]. This recommendation is mainly due to the assumption that glucagon-induced inotropic and chronotropic effects counteract the cardiodepressive effect of beta-blockers. However, this hypothesis still needs to be confirmed because the beneficial effects of glucagon administration could have been due to other treatments that were concomitantly administered. In a recent review that included 119 cases of beta-blocker poisoning, only one of these cases reported haemodynamic improvements after being administered glucagon alone; in the rest of the cases, there was no effect of glucagon, or the beneficial effect may have been due to other treatments with inotropic, chronotropic or vasopressor effects that were concomitantly administered with glucagon [10]. Thus, glucagon is probably not the best drug to administer in the case of cardioinhibitory drug overdose, and other therapies may be more appropriate.

## Supplementary Information

**Additional file 1: Video S1.** Showing a spontaneously beating sample of human sinoatrial node (SN) tissue. The SN is attached to the pectinate muscle (PM), which is connected by a silk thread to an isometric force‒displacement transducer. Contractions of the PM when receiving the stimulus of SN tissue were recorded as the measure of SN rate.

**Additional file 2 Table S2.** Primers used in the study, obtained from Merck, Madrid (Spain).

**Additional file 3 Figure S3.** Inotropic effect of the nonselective phosphodiesterase inhibitor IBMX (30 µM) on electrically driven (1 Hz) trabeculae obtained from isolated right atria (RA), left atria (LA), right ventricles (RV), and left ventricles (LV) of nonfailing (NF) and failing (F) human hearts. Inotropic responses are expressed as percent increases in basal (before the addition of IBMX) contractility. Each column represents the mean value ± SEM (vertical bars) of 5 experiments. *P<0.05, (Wilcoxon test).

**Additional file 4 Figure S4.** Inotropic effect of calcium (9 mM) on electrically driven (1 Hz) trabeculae isolated from right atria (RA), left atria (LA), right ventricle (RV), and left ventricle (LV) from nonfailing (NF) and failing (F) human hearts. Inotropic responses are expressed as percent increases in basal (before the addition of IBMX or glucagon) contractility. Each column represents the mean value ± SEM (vertical bars) of 5 experiments. *P<0.05, (Wilcoxon test).

**Additional file 5 Figure S5.** Chronotropic effect of noradrenaline (NA, 10 μM) and the nonselective phosphodiesterase inhibitor IBMX (30 μM) on spontaneously beating SN tissue obtained from nonfailing (NF) and failing (F) human hearts. The results are expressed as the increase in the basal rate (beats min^**-1**^). Glucagon (1–1000 nM) did not exert chronotropic effects. After washing, IBMX was applied, which increased the beating rate, as shown in the corresponding columns. In the presence of IBMX, glucagon (1–1000 nM) again did not exert chronotropic effects. The experiments were terminated by adding noradrenaline, which further increased chronotropism as indicated. Each bar represents the mean value ± SEM (vertical bars) of 5 experiments. No significant differences were observed between the chronotropic effects of NA and IBMX in samples obtained from F and NF human hearts. P>0.05 (Wilcoxon test).

**Additional file 6 Figure S6.** Western blot analysis showing the absence of glucagon receptor expression in samples of the sinoatrial node (SN), right atrium (RA), left atrium (LA), right ventricle (RV) and left ventricle (LV) from the human heart. Numbers in panels correspond to samples obtained from heart donors in Table 1. α-Tubulin was used as the loading control. Band intensity was determined with densitometry using the program Image Quant TL Plus (General Electrics, USA).

**Additional file 7 Figure S7.** Expression of HCN1 channels in human sinoatrial (SN) node tissue and the right ventricular (RV) myocardium. Protein levels were determined by Western blot analysis using α-Tubulin as the loading control. Band intensity was determined with densitometry using the program Image Quant TL Plus (General Electrics, USA). **A** Representative Western blot showing the expression of HCN1 channels in the SN and RV. **B** Mean values of 10 independent experiments; bars ± SEM (*P<0.05, Wilcoxon test).

## Data Availability

The data used in the current study are available from the corresponding author on reasonable request.

## Abbreviations

cAMP: 3′,5′-Cyclic adenosine monophosphate
PDEs: Phosphodiesterase enzymes
NF: Nonfailing
LA: Left atria
RA: Right atria
RV: Right ventricle
LV: Left ventricle
SN: Sinoatrial node
IBMX: 3-Isobutyl-1-methylxanthine
HCN1: Hyperpolarization-activated cyclic nucleotide-gated 1 channel
PCR: Polymerase chain reaction
WB: Western blotting
NYHA: New York Heart Association
mRNA: Messenger ribonucleic acid
cDNA: Complementary deoxyribonucleic acid
SDS: Sodium dodecyl sulfate
PBS: Phosphate-buffered saline
SEM: Standard error of the mean
