# Supplementary figures and images for: Evidence for a lack of inotropic and chronotropic effects of glucagon and glucagon receptors in the human heart
Source: Cardiovasc Diabetol. 2023 May 30;22:128. doi: 10.1186/s12933-023-01859-8 (PMC10230788; doi:10.1186/s12933-023-01859-8)

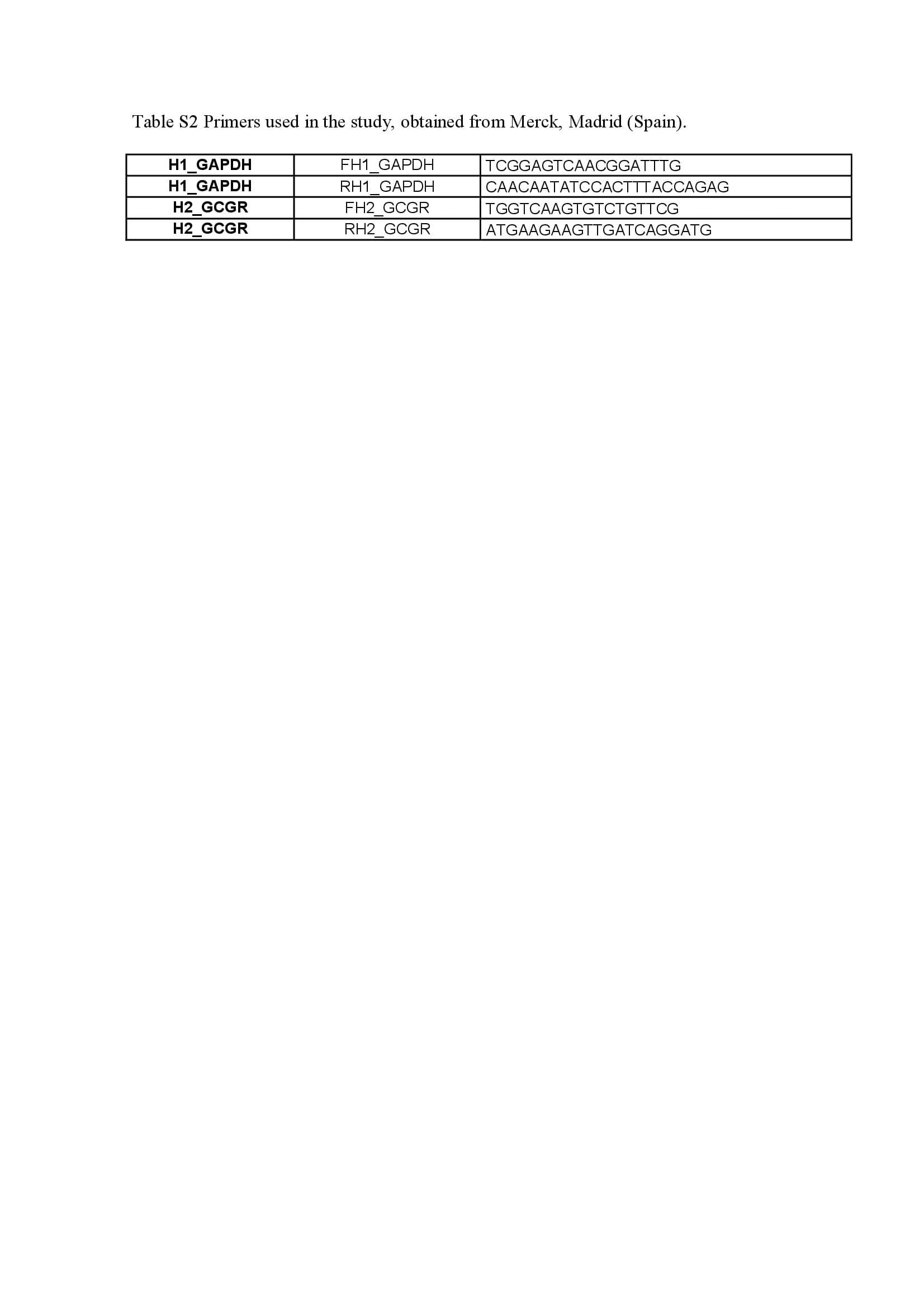

Supplement: Supplementary file 2 — Additional file 2 Table S2. Primers used in the study, obtained from Merck, Madrid (Spain). [file 12933_2023_1859_MOESM2_ESM.jpg]

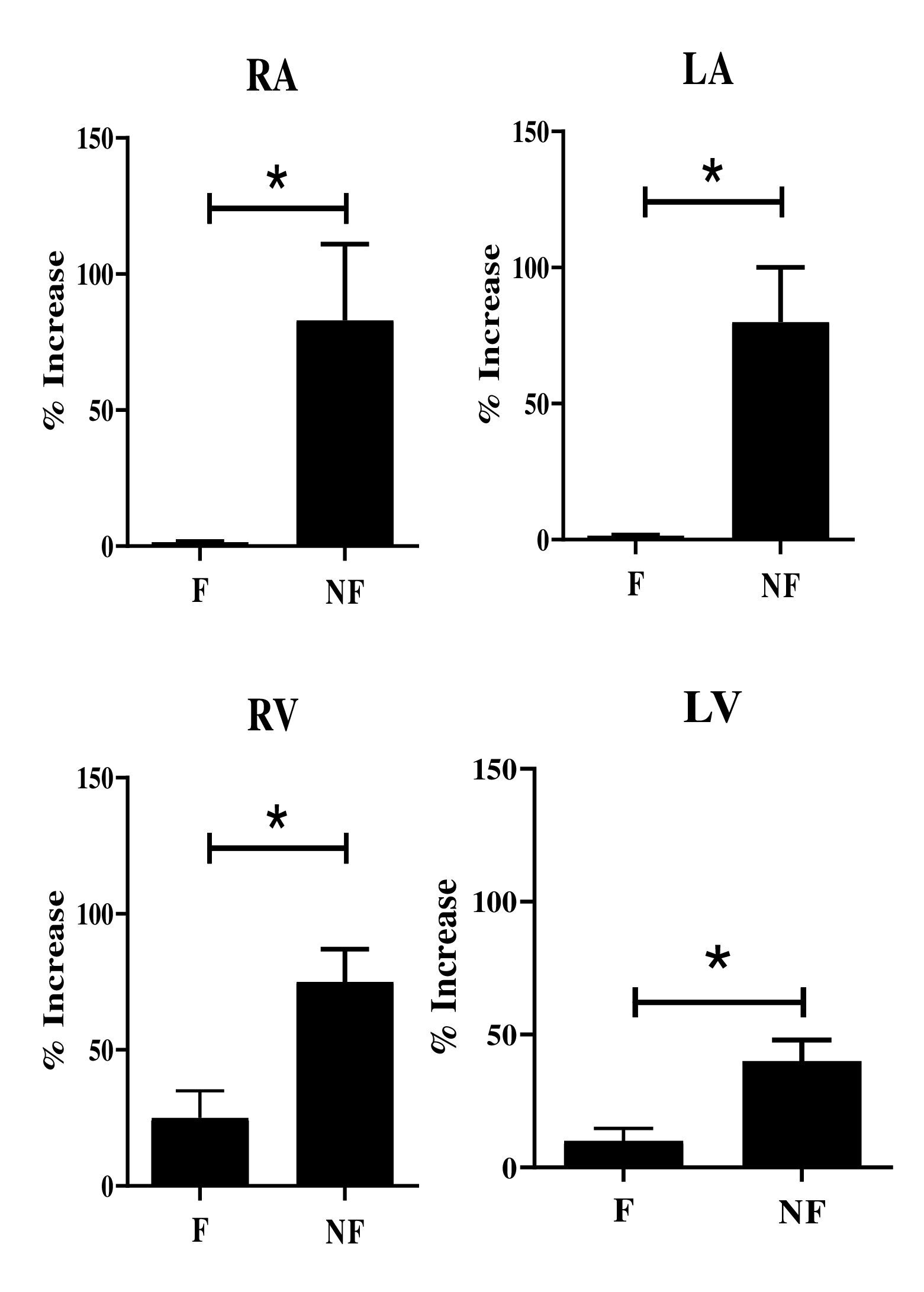

Supplement: Supplementary file 3 — Additional file 3 Figure S3. Inotropic effect of the nonselective phosphodiesterase inhibitor IBMX (30 µM) on electrically driven (1 Hz) trabeculae obtained from isolated right atria (RA), left atria (LA), right ventricles (RV), and left ventricles (LV) of nonfailing (NF) and failing (F) human hearts. Inotropic responses are expressed as percent increases in basal (before the addition of IBMX) contractility. Each column represents the mean value ± SEM (vertical bars) of 5 experiments. *P<0.05, (Wilcoxon test). [file 12933_2023_1859_MOESM3_ESM.jpg]

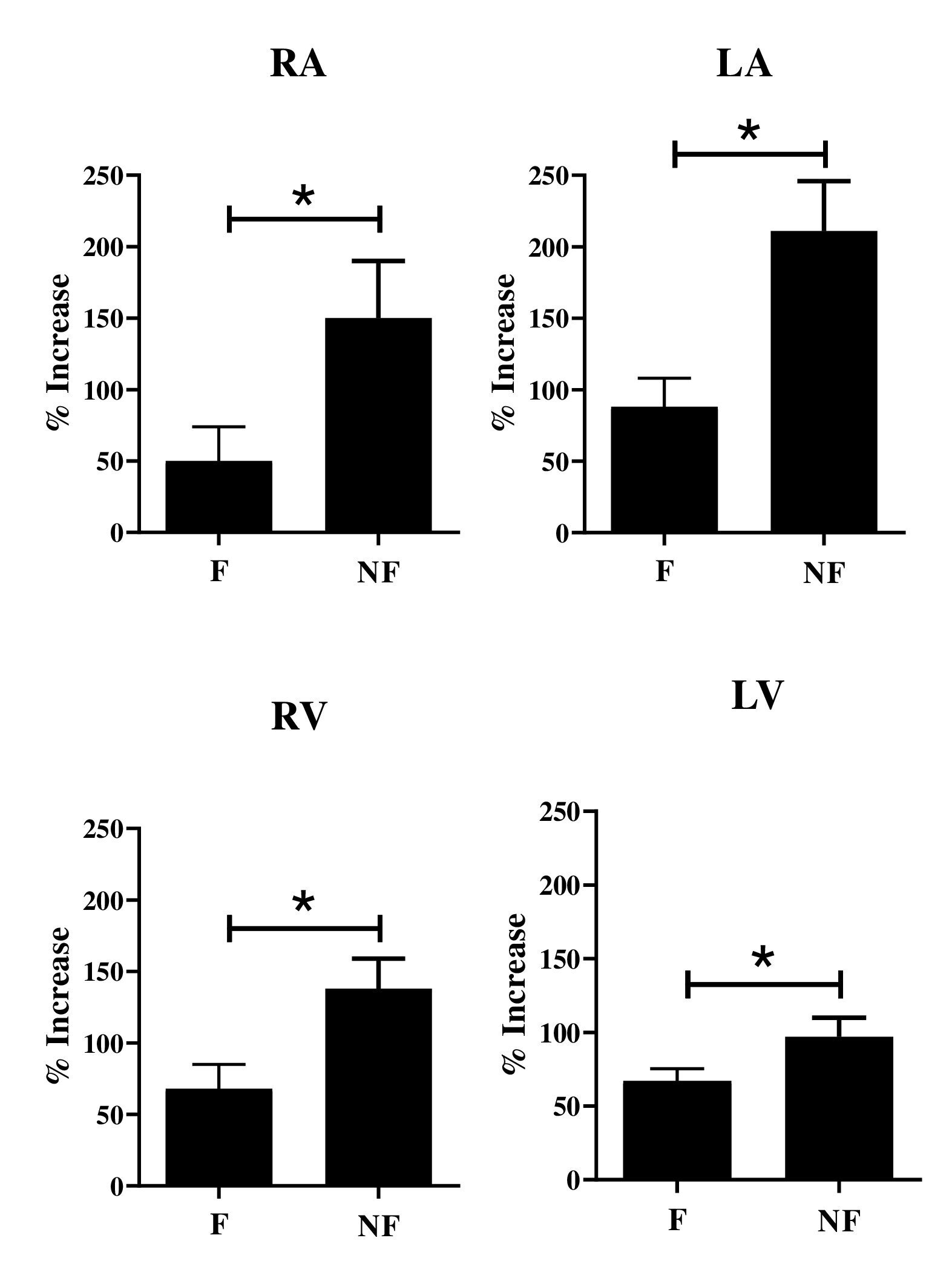

Supplement: Supplementary file 4 — Additional file 4 Figure S4. Inotropic effect of calcium (9 mM) on electrically driven (1 Hz) trabeculae isolated from right atria (RA), left atria (LA), right ventricle (RV), and left ventricle (LV) from nonfailing (NF) and failing (F) human hearts. Inotropic responses are expressed as percent increases in basal (before the addition of IBMX or glucagon) contractility. Each column represents the mean value ± SEM (vertical bars) of 5 experiments. *P<0.05, (Wilcoxon test). [file 12933_2023_1859_MOESM4_ESM.jpg]

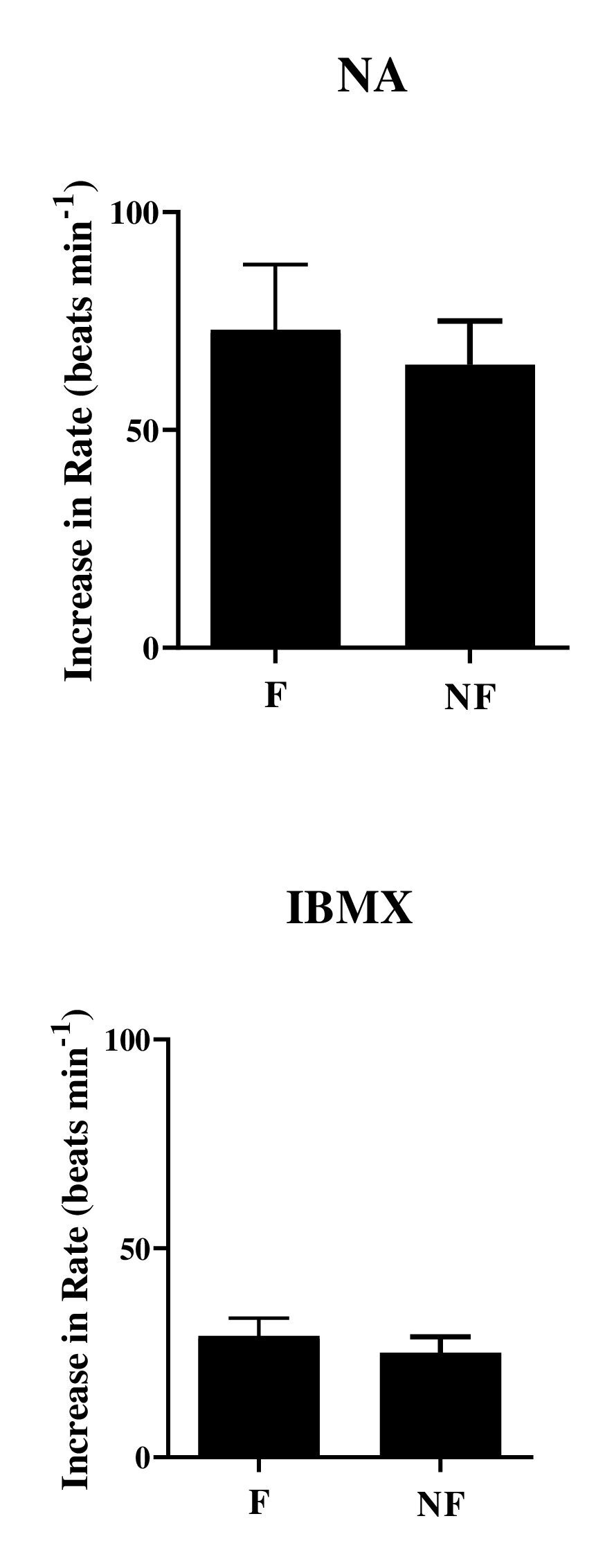

Supplement: Supplementary file 5 — Additional file 5 Figure S5. Chronotropic effect of noradrenaline (NA, 10 μM) and the nonselective phosphodiesterase inhibitor IBMX (30 μM) on spontaneously beating SN tissue obtained from nonfailing (NF) and failing (F) human hearts. The results are expressed as the increase in the basal rate (beats min-1). Glucagon (1–1000 nM) did not exert chronotropic effects. After washing, IBMX was applied, which increased the beating rate, as shown in the corresponding columns. In the presence of IBMX, glucagon (1–1000 nM) again did not exert chronotropic effects. The experiments were terminated by adding noradrenaline, which further increased chronotropism as indicated. Each bar represents the mean value ± SEM (vertical bars) of 5 experiments. No significant differences were observed between the chronotropic effects of NA and IBMX in samples obtained from F and NF human hearts. P>0.05 (Wilcoxon test). [file 12933_2023_1859_MOESM5_ESM.jpg]

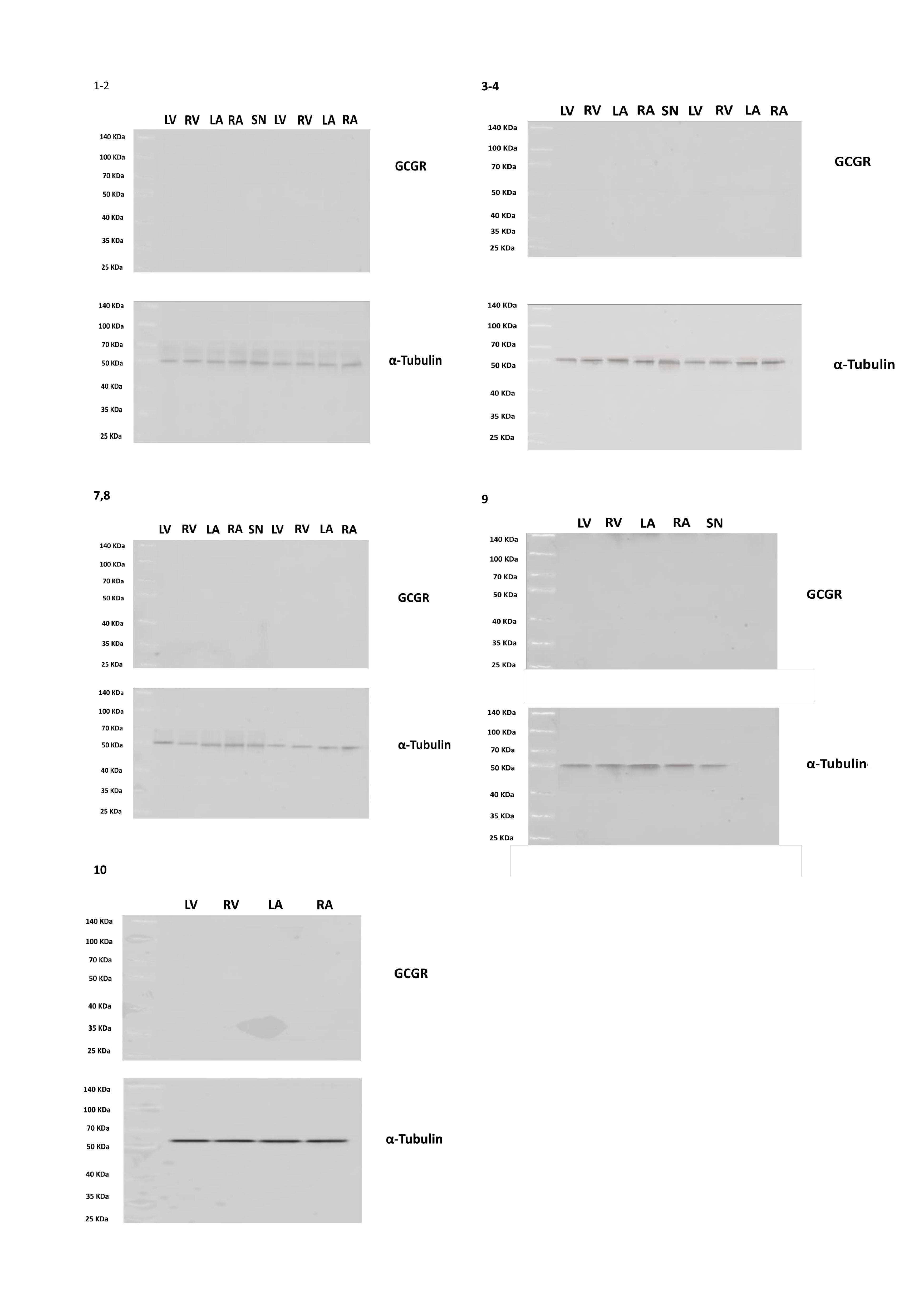

Supplement: Supplementary file 6 — Additional file 6 Figure S6. Western blot analysis showing the absence of glucagon receptor expression in samples of the sinoatrial node (SN), right atrium (RA), left atrium (LA), right ventricle (RV) and left ventricle (LV) from the human heart. Numbers in panels correspond to samples obtained from heart donors in Table 1. α-Tubulin was used as the loading control. Band intensity was determined with densitometry using the program Image Quant TL Plus (General Electrics, USA). [file 12933_2023_1859_MOESM6_ESM.jpg]

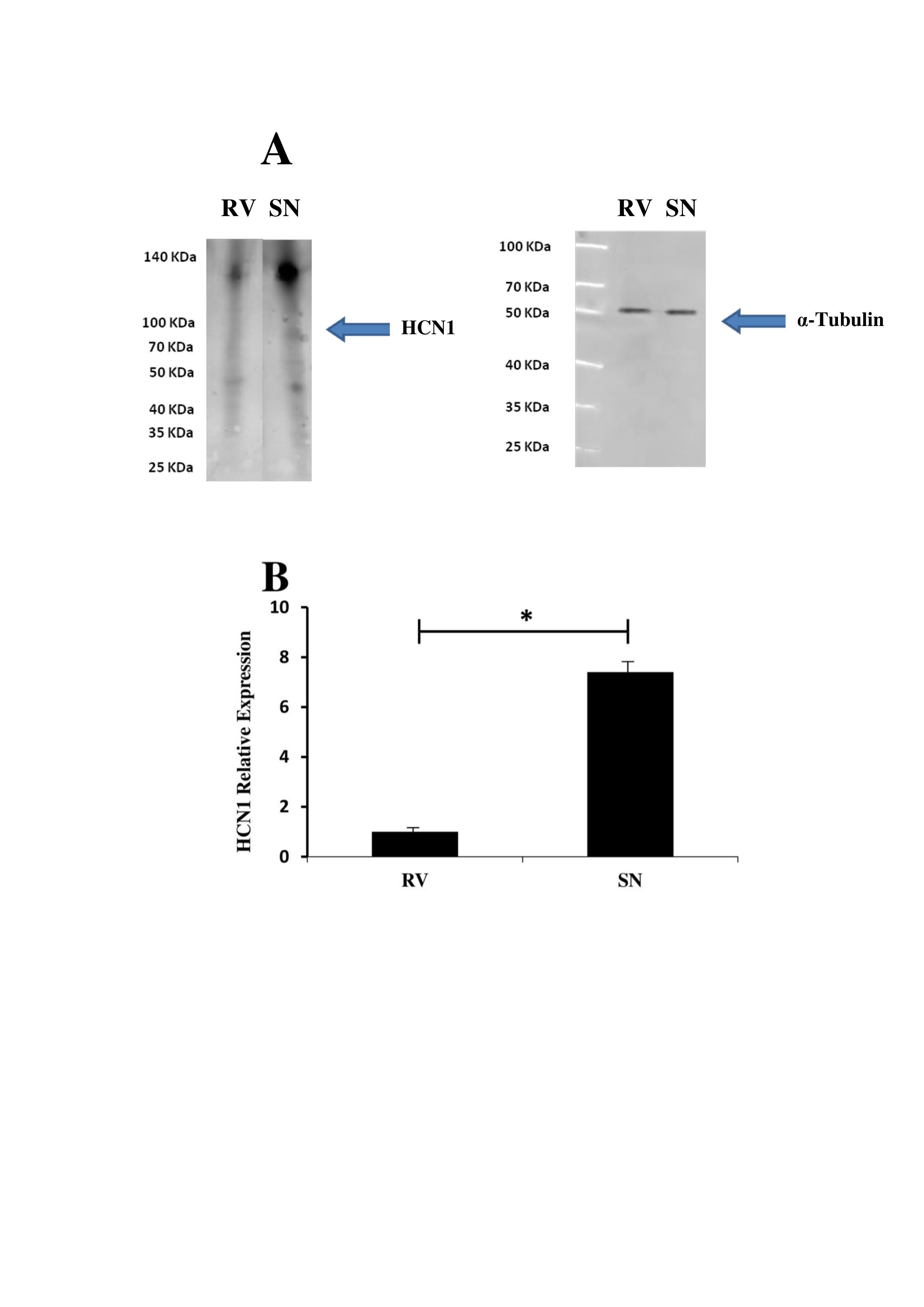

Supplement: Supplementary file 7 — Additional file 7 Figure S7. Expression of HCN1 channels in human sinoatrial (SN) node tissue and the right ventricular (RV) myocardium. Protein levels were determined by Western blot analysis using α-Tubulin as the loading control. Band intensity was determined with densitometry using the program Image Quant TL Plus (General Electrics, USA). A Representative Western blot showing the expression of HCN1 channels in the SN and RV. B Mean values of 10 independent experiments; bars ± SEM (*P<0.05, Wilcoxon test). [file 12933_2023_1859_MOESM7_ESM.jpg]
